# Healthcare-related impact of gout in hospitalized patients in Spain

**DOI:** 10.1038/s41598-021-92673-3

**Published:** 2021-06-24

**Authors:** Diego Benavent, Diana Peiteado, María Ángeles Martinez-Huedo, María Hernandez-Hurtado, Alejandro Balsa, Eugenio de Miguel

**Affiliations:** 1 Rheumatology Service, Hospital Universitario La Paz-IdiPaz, Madrid, Spain; 2 Preventive Medicine and Public Health Teaching and Research Unit, Hospital Universitario La Paz , Madrid, Spain; 3 General Practice and Community Medicine Unit, Hospital Fundación Alcorcón, Madrid, Spain

**Keywords:** Crystal deposition arthropathies, Gout, Epidemiology, Health care economics

## Abstract

To analyze the epidemiology, clinical features and costs of hospitalized patients with gout during the last decade in Spain. Retrospective observational study based on data from the Minimum Basic Data Set (MBDS) from the Spanish National Health Service database. Patients ≥ 18 years with any gout diagnosis at discharge who had been admitted to public or private hospitals between 2005 and 2015 were included. Patients were divided in two periods: p1 (2005–2010) and p2 (2011–2015) to compare the number of hospitalizations, mean costs and mortality rates. Data from 192,037 patients with gout was analyzed. There was an increase in the number of hospitalized patients with gout (p < 0.001). The more frequent comorbidities were diabetes (27.6% of patients), kidney disease (26.6%) and heart failure (19.3%). Liver disease (OR 2.61), dementia (OR 2.13), cerebrovascular diseases (OR 1.57), heart failure (OR 1.41), and kidney disease (OR 1.34) were associated with a higher mortality risk. Women had a lower risk of mortality than men (OR 0.85). General mortality rates in these hospitalized patients progressively increased over the years (p < 0.001). In addition, costs gradually rose, presenting a significant increase in p2 even after adjusting for inflation (p = 0.001). A progressive increase in hospitalizations, mortality rates and cost in hospitalized patients with gout was observed. This harmful trend in a preventable illness highlights the need for change and the search for new healthcare strategies.

## Introduction

Gout is a common form of inflammatory arthritis that leads to substantial morbidities and impairs patients’ quality of life. It is caused by the deposition of monosodium urate crystals within the joints and other tissues^1^, producing attacks with significant pain, with increasing evidence linking it to subclinical inflammation, cardiovascular risk and associated comorbidities^2–4^.

Today, monosodium urate crystal formation can be effectively avoided with urate-lowering therapy. However, the evidence indicates that this effective treatment is not provided for a majority of patients^5^. This lack of treatment effectiveness is most apparent in the number of patient hospitalizations due to gout attacks or comorbidities.

According to recent research, there is an increasing trend in the prevalence of gout in Western countries^6^. Thus, the prevalence of gout in the UK in 1991 had increased threefold compared with estimates from the 1970s^7^, and similar trends were confirmed in several more recent studies in other nations^8,9^. Although reasons for this are unknown, some risk factors, longer life expectancy and increased awareness of the disease among health professionals have been indicated as potential contributing factors^10^.

If this holds true, health services should develop strategies to improve the diagnosis and treatment of this disease. To determine the impact of gout during the last decade in our country, our main objective was to analyze the epidemiology, clinical features and costs of hospitalized patients with gout during the last decade in Spain.

## Methods

### Study population

Retrospective observational study based on data from the Minimum Basic Data Set (MBDS) from the Spanish National Health Service database, which includes hospitalizations in the Spanish population.

The MBDS contains demographic data, diagnoses, comorbidities and complications presented by patients, as well as the diagnostic techniques and surgical procedures employed during their hospitalization. Diagnoses are coded according to the International Classification of Diseases (9th Revision, Clinical Modification-ICD-9-CM), which allows for classifying the episodes into diagnostic-related groups (DRGs).

Data was requested from the Ministry of Health; specifically, access to the Discharge Hospital Registry of the MBDS for the pertinent information on patients ≥ 18 years with any gout diagnosis at discharge who had been admitted to public or private hospitals between 2005 and 2015. The requested data included the following diagnostic codes as a primary or secondary diagnosis using International Classification of Diseases-9 (ICD-9): 274.0: gout arthropathy. 274.00: gout arthropathy, unspecified. 274.01: Gout arthropathy, acute. Gout attack. Podagra. 274.02: Chronic gout arthropathy without tophi. 274.03: Chronic gout arthropathy with tophi/Chronic tophaceous gout. 274.1: Gout nephropathy. 274.10: Gout nephropathy, not specified. 274.11: Gout nephrolithiasis. 274.19: Other. 274.8: Gout with other specified manifestations. 274.81: Tophi on the ear. 274.82: Tophi on other sites. Tophi in the heart. 274.89: Gout with other manifestations. 274.9: Gout, non-specified.

Regarding the general inpatient population, we obtained data on the total number of discharges, the mean duration of the hospital stays, overall in-hospital mortality and costs for the entire population included in the MBDS.

Patients were divided in two periods of time: period 1 (p1), which comprises years 2005–2010; and period 2 (p2), including years 2011–2015. Total number of hospitalizations, mean costs and mortality rates were compared between both groups.

In addition, the presence of comorbidities such as diabetes, congestive heart failure (CHF), acute myocardial infarction (AMI), cerebrovascular disease (CVD), liver disease, dementia and kidney disease were identified by ICD-9 MC codes for the analysis of this study.

As a secondary objective, a sub-analysis of patients discharged with a main diagnosis of gout was also carried out.

### Statistical analysis

The full cohort of patients with a primary or secondary diagnosis of gout at discharge was analyzed. Mean and standard deviation were used for the description of continuous quantitative variables. Qualitative variables are described by absolute frequencies and relative frequencies are expressed as a percentage.

Comparisons between continuous quantitative variables were performed using the Student t-test for independent groups. The analysis of frequencies between qualitative variables was carried out using the Chi-squared test (χ^2^) or the exact Fisher test (if N < 20, or if any value in the table of expected values was less than 5).

In order to identify factors independently associated with mortality in these patients, a multivariate analysis was performed using forward stepwise logistic regression based on the improvement of the Likelihood Ratio (LR). The possible predictors were introduced as independent variables, including age, gender, diabetes, CHF, CVD, AMI, liver disease, dementia and kidney disease, and mortality as the dependent variable. The magnitude of its adjusted effect was expressed by the odds ratio and 95% of its confidence interval (CI).

SPSS software version 21 was used to analyze the data. Statistically significant differences were considered for an error probability of less than 5% (p < 0.05).

## Results

The study cohort consisted of 192,037 patients with a discharge diagnosis of gout during the period 2005–2015, among the 40,397,139 total discharges during the same period in the National Health System. This means that the 0.48% of hospitalization discharges in our country presented gout among the discharge diagnosis; in 10,512 cases gout was the primary cause of the hospitalization, and in 181,525 cases it appeared as part of another diagnosis.

**Figure 1:**
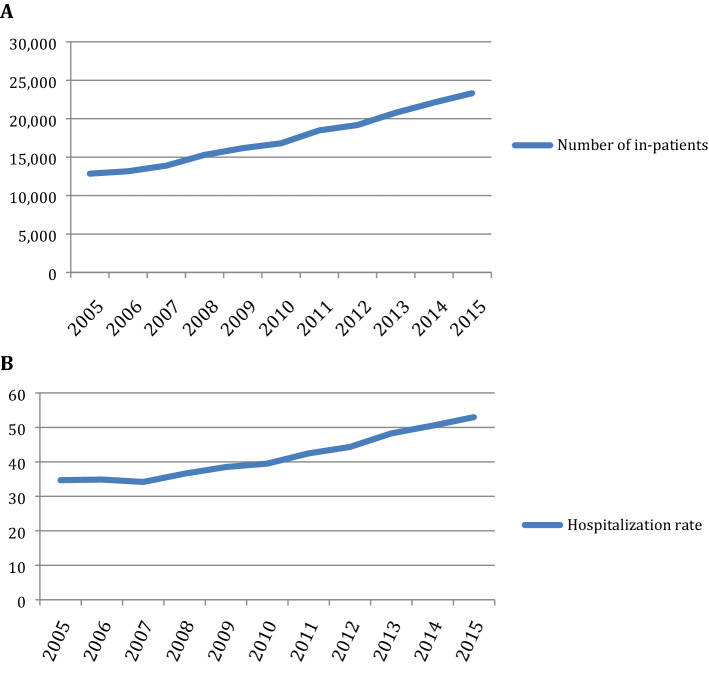
**(A)** Number of hospitalizations by year in Spain. **(B)** Hospitalization rate per 10,000 hospitalizations by year in the MBDS.

In the study population, 158,646 were males (82.6%) and total mean age at hospitalization was 72.1 ± 12.6 years. The number of hospitalizations, as assessed by discharge diagnosis, increased each year (Fig. 1). Concerning the outcome of the hospitalization, 91.6% (175,948) were discharged from hospital, 3.6% (6,870) were transferred to another center, and 4.3% (8,179) died. The average stay decreased during the time of the study from 11 days in 2005 to 8.9 days in 2015 (Table 1), which yielded statistically significant differences between p1 and p2 (p < 0.001).

**Table 1:** Mean hospital stays and in-hospital mortality for gout in Spain.

Year	Mean hospital stay (mean ± SD)	Mortality (%)	
2005	11.00 (12.25)		532 (4.1%)
2006	10.63 (10.81)		479 (3.6%)
2007	10.46 (10.6)		544 (3.9%)
2008	10.58 (11.17)		601 (3.9%)
2009	10.33 (11.02)		630 (3.9%)
2010	9.96 (10.37)		658 (3.9%)
2011	9.62 (9.86)		781 (4.2%)
2012	9.21 (10.27)		897 (4.7%)
2013	9.09 (9.42)		926 (4.5%)
2014	8.94 (9.37)		991 (4.5%)
2015	8.90 (9.77)		1139 (4.9%)
Total	9.74(10.37)		8,178(4.3%)

The main comorbidities in these patients were DM2 (27.6% of the study population), kidney disease (26.6%), CHF (19.3%), AMI (7.4%), CVD (6.7%), liver disease (2.7%) and dementia (1.4%) (Table 2).

**Table 2:** Comorbidities associated in gout as main diagnosis and gout as any diagnosis.

Disease	% of patients (any gout diagnosis)	% of patients (gout as primary diagnosis)
Diabetes	27.6	19.9
Kidney failure	26.6	20
CHF	19.3	4.4
AMI	7.4	2.7
CVD	6.7	3.2
Liver disease	2.7	1.4
Dementia	1.4	0.8

*CHF* congestive heart failure, *AMI* acute myocardial infarction, *CVD* cerebrovascular disease.

Regarding mortality during hospitalization, the percentages progressively increased, reaching the highest rates in 2015 with 4.9% (Table 1), with statistically significant differences between p1 and p2 mortality rates (p < 0.001) The mean age of death was 77.3 years in males and 83.1 years in females (p < 0.01).

The multivariate analysis revealed factors associated with risk of mortality related to gout. Hence, in the study population, women had a lower risk of mortality (OR 0.85, 95% CI 0.80–0.90) than men, and older age was associated with a greater risk of mortality (OR 1.06, 95% CI 1.05–1.06). Other conditions that were associated with an increased mortality were: CVD (OR 1.57, CI 95% 1.46–1.49), CHF (OR: 1.41, CI 95% 1.34–1.49), liver disease (OR 2.61, CI 95% 2.34–2.9), kidney disease (OR 1.34, CI 95% 1.28–1.41) and dementia (OR 2.13, CI 95% 1.88–2.42). In the case of type DM2, a significantly lower risk of mortality was observed (OR 0.92, CI 95%, 0.87–0.96).

The average cost increased over time, with a minimum of 4,219 €/stay in 2005 and a maximum of 5,384 €/stay in the last year of analysis (Table 3). Mean costs per patient in p2 were higher than in p1 (5232.40 versus 4664.50, p = 0.02). Moreover, these costs are 9% higher than the mean cost for medical hospitalization in the MBDS. When adjusting for retail price index (RPI), these differences disappeared (p = 0.78). However, when comparing total costs per year (in millions of euros [€]), costs in p2 where significantly higher than p1 even after RPI adjustment (109,520 versus 77,050, p = 0.001).

**Table 3:** Annual costs of hospitalization in gout patients in Spain.

Year	Mean cost per patient € (mean ± SD)	Mean cost per patient RPI adjusted*	Cost/year millions €	Cost/year millions € RPI adjusted
2005	4219 (3513)	5113	54.2	65.7
2006	4269 (4188)	4964	56.2	65.3
2007	4459 (4083)	5065	61.6	69.9
2008	4838 (4491)	5268	74	80.6
2009	4997 (4605)	5396	80.8	87.3
2010	5205 (5537)	5564	87.5	93.5
2011	5285 (5930)	5480	99.5	103.1
2012	5247 (5357)	5325	100.6	102.1
2013	5166 (5318)	5109	107.3	106.1
2014	5080 (4810)	5013	112.3	110.8
2015	5384 (4908)	5384	125.5	125.5
Total	4999 (4935)	–	959.5	–

*RPI* retail price index.

*Adjusted in relationship to 2015.

A continuous and linear annual increase in the number of patients with any discharge diagnosis of gout was observed from 2005 (12,851 patients) to 2015 (23,320), with a mean of 14,696 patients per year in p1 and 20,772 in p2 (p < 0.001).

Regarding gout as a primary diagnosis, among the 192,037 discharged patients, 10,512 met this condition in the whole period. Of these, 85.9% were male, and mean age at hospitalization was 65.4 ± 14.7 years. 4555 (43.4%) patients were at least 70 years old. By gender, mean age was 64.02 ± 14.43 years in males and 73.9 ± 13.69 years in females (p < 0.001). The main comorbidities in these patients were kidney disease (20.0%), DM2 (19.9%) and CHF (4.4%). Some other comorbidities that were investigated in these patients were: AMI (2.7%), CVD (3.2%), liver disease (1.4%) and dementia (0.8%). All these comorbidities were significantly lower (p < 0.05) in this group than in the group of any gout diagnosis. Among all patients with gout as primary diagnosis, 62.6% of males and 45.6% of women did not show any comorbidity (p < 0.05). 98.0% of patients (10,236) were discharged, 1.5% (156) were transferred to another center, and 0.5% (56) died. Considering mean age at death, there were no statistically significant differences in gender distribution in this group (males 76.58 ± 11.13 years and females 76.9 ± 13.8 years). The mean stay of these patients was 6.71 ± 6.8 days with an average cost of 3,471 ± 2,678€. Mean cost per patient and year of treatment in those patients with gout as primary diagnosis was higher in p2 than in p1 (3971€ versus 2983€, p < 0.001) (Supplementary Fig. 1).

Three specialties attended 80.6% of the patients with gout as main discharge diagnosis: Internal Medicine, with 3852 admissions (36.6%), Rheumatology, with 2600 (24.7%) and Orthopedics, with 2,033 (19.3%); the average number of patients per year was 350, 236 and 185, respectively. Mean length of stay of patients was shorter in Orthopedics, with a mean of 4.85 ± 6.78 days, followed by Rheumatology (6.52 ± 5.65) days and Internal Medicine (7.76 ± 6.83 days). The cost was lower in patients admitted by the Rheumatology service (Supplementary Table 1).

Multivariate analyses revealed factors associated with an increased risk of mortality in the group of gout as primary diagnosis: CHF (OR 3.39, CI 95% 1.74–6.63), AMI (OR 3.34, CI 95% 1.41–7.91), and older age (OR 1.06, CI 95% 1.03–1.08). Patients with gout as a primary diagnosis were younger (p < 0.05) and had less comorbidity than patients in the full study population (p < 0.05) (Table 2).

## Discussion

This observational study collected data on patients discharged with a diagnosis of gout during 2005–2015 in hospitals of the Spanish National Health Service, and analyzed characteristics of their hospitalizations. There was a steady increase in the number of admissions with gout during the study period, with an annual increase of 5.4% over 10 years. These data are consistent with previous publications^11,12^. Rai et al*.* reported a twofold increase in gout hospitalizations from 2000 to 2011 in the Canadian population, doubling gout costs over the same study period^13^; this figure is similar to our results. This upward trend was not observed in the MBDS of Spanish national hospitalizations^14^. In addition, the average stay for hospitalized gouty patients was 9.7 days, which was higher (p < 0.01) than the average hospitalization for the general population (mean of 7.2 days for medical conditions during the same period)^14^. Analyzing this increase in the length of hospitalizations in gout patients, we hypothesize that the main contributing risk factors might be the presence of comorbidities, such as DM2 or kidney failure.

Our study reveals that in-patients with gout have a high burden of comorbidities. The comorbidities that showed stronger associations with gout were DM2 (27.6%), CHF (19.3%) and renal disease (26.6%). It is well known that these comorbidities produce an increase in the rates of morbidity and mortality, with a corresponding impact on the use of resources and their associated costs^15^. Certain components of metabolic syndrome and renal disease are associated with gout and gout duration^16^. Regarding DM2, its relationship with gout is more controversial; individuals with DM2 have a lower risk of developing gout, but patients with gout have an increased risk of developing diabetes^17^. Cardiovascular diseases have been widely described in patients with gout^18^, and there is growing experimental evidence that supports an effect of high concentrations of urate on endothelial dysfunction^19^. Regarding renal disease, it has been reported that 20% of adults with gout have chronic kidney disease ≥ stage 4 and above^20^. Interestingly, Singh et al. found that renal failure, heart failure, and diabetes were associated with a longer hospital stay for a hospitalization due to gout in a multivariable analysis^21^.

Regarding mortality, around 4–5% of the study patients with a diagnosis of gout died during their hospitalization. Mortality increased from 2005 to 2015 in both absolute and relative terms (4.1–4.9%, equating to a 0.8% increase), whereas mortality for any cause in the MBDS increased only 0.4% (3.3–3.7%)^14^. This lower death rate for other medical problems suggests that gout, with its higher risk of mortality, warrants the development of new health strategies to reverse this trend. This conclusion was reached in previous studies, notably a systematic review that encompassed seven long-term studies^22^. Lottmann et al*.* observed that the increased mortality risk in gouty patients was around 1.2–1.8 higher compared to those without gout. This risk was particularly true of patients with comorbidities. These results are similar to those presented in our study. It is also interesting that the mean age of death in patients with gout was lower than expected (77.3 years in males and 83.1 years in females), being significantly lower than that of general population (78.8 and 84.8 years for males and females, respectively, during the same period of time)^14^ (p < 0.01). This difference is especially pronounced in female gout patient subgroup with gout as the main discharge diagnosis, where the mean age at death of women was 76.9 years without difference with males and with difference of almost 6–8 years lower compared to the expected age of death in females the general population. This data highlights that we must pay special attention to female gout.

Studies on the costs of gout patient hospitalizations remain very scarce^23^. Our results showed an increase in crude costs per patient that disappeared when adjusted by the rate of inflation, probably due to reduction in the hospitalization average stage. However, since the number of gouty patients has risen, there has been a two-fold increase in costs even after adjusting for inflation.

An important strength of our study is its large population-based dataset, with over 10 years of follow-up; the MBDS provides an accurate compilation of all hospitalizations in our country. As shown in previous studies, the MBDS has proven to be reliable and valuable for descriptive and evaluative purposes^24^. Some limitations should be noted. Firstly, some data are not reported in the hospitalized patients in our cohort, such as treatment or blood test results that are not available in the MBDS. In addition, the study population included patients who were admitted to a hospital for any reason, when gout might have been the only comorbidity. Furthermore, costs are based on the average cost per hospitalized bed-day, which should be taken into consideration when assessing these data. Finally, the validity of gout as a discharge diagnosis in the Spanish National Hospital Discharge Database has not been evaluated. However, Singh et al. found an accuracy of administrative codes with ICD-9 for gout that yielded 86% sensitivity (95% CI, 78%-96%), 95% specificity (95% CI, 91%-100%), 86% positive predictive value, and 95% negative predictive value. They concluded that administrative codes can be used for health services outcomes research in gout^25^.

In conclusion, we observed a progressive increase in hospitalizations, cost and mortality rates in hospitalized patients with gout over the study period. This worrisome trend in a preventable illness highlights the need for change and the search for new healthcare strategies.

## Supplementary Information

Supplementary Table 1.

Supplementary Figure 1.

## References

[1] Harris MD, Siegel LB, Alloway JA (1999). Gout and hyperuricemia. Am. Fam. Phys..

[2] Choi HK, Curhan G (2007). Independent impact of gout on mortality and risk for coronary heart disease. Circulation.

[3] Krishnan E, Baker JF, Furst DE, Schumacher HR (2006). Gout and the risk of acute myocardial infarction. Arthritis Rheum..

[4] Neogi T, Hunter DJ, Chaisson CE, Allensworth-Davies D, Zhang Y (2006). Frequency and predictors of inappropriate management of recurrent gout attacks in a longitudinal study. J. Rheumatol..

[5] Roddy E, Zhang W, Doherty M (2007). Concordance of the management of chronic gout in a UK primary-care population with the EULAR gout recommendations. Ann. Rheum. Dis..

[6] Zhu Y, Pandya BJ, Choi HK (2011). Prevalence of gout and hyperuricemia in the US general population: The National Health and Nutrition Examination Survey 2007–2008. Arthritis Rheum..

[7] Harris CM, Lloyd DCEF, Lewis J (1995). The prevalence and prophylaxis of gout in England. J. Clin. Epidemiol..

[8] Dehlin M, Jacobsson L, Roddy E (2020). Global epidemiology of gout: Prevalence, incidence, treatment patterns and risk factors. Nat. Rev. Rheumatol..

[9] Singh G, Lingala B, Mithal A (2019). Gout and hyperuricaemia in the USA: Prevalence and trends. Rheumatology (Oxford)..

[10] Zobbe K, Prieto-Alhambra D, Cordtz R, Højgaard P, Hindrup JS, Kristensen LE, Dreyer L (2019). Secular trends in the incidence and prevalence of gout in Denmark from 1995 to 2015: A nationwide register-based study. Rheumatology (Oxford)..

[11] Blakey G, Callear J (2019). Gout in primary care: Can we improve patient outcomes?. Br. J. Gen. Pract..

[12] Robinson PC, Merriman TR, Herbison P, Highton J (2013). Hospital admissions associated with gout and their comorbidities in New Zealand and England 1999–2009. Rheumatology (United Kingdom)..

[13] Rai SK, Aviña-Zubieta JA, McCormick N, de Vera MA, Lacaille D, Sayre EC (2017). Trends in gout and rheumatoid arthritis hospitalizations. Arthritis Care Res..

[14] Msssi.gob.es [Internet]. *Ministerio**de**Sanidad,**Consumo**y**Bienestar**Social,**MBDS**[Conjunto**Mínimo**Básico**Datos]*. https://www.msssi.gob.es/estadEstudios/estadisticas/inforRecopilaciones/anaDesarrolloGDR.htm. (2016).

[15] Elfishawi MM, Zleik N, Kvrgic Z, Michet CJ, Crowson CS, Matteson EL (2018). The rising incidence of gout and the increasing burden of comorbidities: A population-based study over 20 years. J. Rheumatol..

[16] Bardin T, Richette P (2017). Impact of comorbidities on gout and hyperuricaemia: An update on prevalence and treatment options. BMC Med..

[17] Pan A, Teng GG, Yuan JM, Koh WP (2016). Bidirectional association between diabetes and gout: The Singapore Chinese Health Study. Sci. Rep..

[18] Singh JA, Gaffo A (2020). Gout epidemiology and comorbidities. Semin. Arthritis Rheum..

[19] Mercuro G, Vitale C, Cerquetani E, Zoncu S, Deidda M, Fini M, Rosano GM (2004). Effect of hyperuricemia upon endothelial function in patients at increased cardiovascular risk. Am. J. Cardiol..

[20] Roughley M, Sultan AA, Clarson L, Muller S, Whittle R, Belcher J (2018). Risk of chronic kidney disease in patients with gout and the impact of urate lowering therapy: A population-based cohort study. Arthritis Res. Ther..

[21] Singh JA, Yu S (2016). Gout-related inpatient utilization: A study of predictors of outcomes and time trends. Arthritis Res. Ther..

[22] Lottmann K, Chen X, Schädlich PK (2012). Association between gout and all-cause as well as cardiovascular mortality: A systematic review. Curr. Rheumatol. Rep..

[23] Wu EQ, Forsythe A, Guerin A, Yu AP, Latremouille-Viau D, Tsaneva M (2012). Comorbidity burden, healthcare resource utilization, and costs in chronic gout patients refractory to conventional urate-lowering therapy. Am. J. Ther..

[24] Peña-Rey I, Martínez de Aragón MV, Villaverde Hueso A (2009). Epidemiología de la varicela en España en los períodos pre y post vacunación. Rev. Esp. Salud Pública.

[25] Singh JA (2013). Veterans Affairs databases are accurate for gout-related health care utilization: A validation study. Arthritis Res. Ther..

