# Supplementary material for: Healthcare-related impact of gout in hospitalized patients in Spain
Source: Sci Rep. 2021 Jun 24;11:13287. doi: 10.1038/s41598-021-92673-3 (PMC8225766; doi:10.1038/s41598-021-92673-3)
Supplement: Supplementary file 1 — Supplementary Table 1. [file 41598_2021_92673_MOESM1_ESM.docx]

**Supplementary Table 1**. Median length of stay and costs of patients admitted for gout as main diagnosis in Internal Medicine, Rheumatology and Orthopedics departments

|  | **Internal Medicine** | **Rheumatology** | **Orthopedics** |
| --- | --- | --- | --- |
| *n* (%) | 3,852 (36.6) | 2,600 (24.7) | 2,033 (19.3) |
| Mean stay (SD), days | 7.76 (6.83) | 6.52 (5.65) | 4.85 (6.78) |
| Mean cost (SD), € | 3,548 (1,796) | 2,891 (1,805) | 3,715 (2,541) |
