# Supplementary material for: Healthcare-related impact of gout in hospitalized patients in Spain
Source: Sci Rep. 2021 Jun 24;11:13287. doi: 10.1038/s41598-021-92673-3 (PMC8225766; doi:10.1038/s41598-021-92673-3)
Supplement: Supplementary file 2 — Supplementary Figure 1. [file 41598_2021_92673_MOESM2_ESM.docx]

Supplementary Figure 1: Mean cost per patient and year of treatment in patients with gout as primary diagnosis.
